# Availability of diagnostic services and essential medicines for non-communicable respiratory diseases in African countries

**DOI:** 10.5588/ijtld.20.0762

**Published:** 2021-02-01

**Authors:** C. Plum, M. Stolbrink, L. Zurba, K. Bissell, B. O. Ozoh, K. Mortimer

**Affiliations:** 1 Liverpool School of Tropical Medicine, Liverpool; 2 Institute of Infection,Veterinary and Ecological Sciences, Liverpool, UK; 3 Education for Health Africa, Durban, South Africa; 4 School of Population Health, University of Auckland, Auckland, New Zealand; 5 College of Medicine, University of Lagos, Lagos, Nigeria

**Keywords:** spirometry, asthma, COPD

## Abstract

**BACKGROUND:**

The global burden of disease due to asthma and chronic obstructive pulmonary disease (COPD) is substantial and particularly great in low- and middle-income countries, including many African countries. Management is affected by availability of diagnostic tests and essential medicines. The study aimed to explore the availability of spirometry services and essential medicines for asthma and COPD in African countries.

**METHOD:**

Questionnaires were delivered to healthcare workers at the annual meeting of the Pan African Thoracic Society Methods in Epidemiology and Clinical Research (PATS MECOR) and International Multidisciplinary Programme to Address Lung Health and TB in Africa (IMPALA). Data were analysed using simple descriptive statistics.

**RESULTS:**

A total of 37 questionnaires representing 13 African countries were returned. Spirometry availability was 73.0%. The most common reasons for non-availability were lack of knowledge of the utility of the test. Within the study sample, 33.3% faced sporadic availability due to maintenance issues. Essential medicines availability ranged from 37.8% for inhaled corticosteroid-long-acting beta-agonist inhalers to 100% for prednisolone 5 mg tablets, mainly due to supply chain problems.

**CONCLUSION:**

There is varied availability of spirometry and WHO essential medicines for COPD and asthma in African countries. Strategies are needed to improve access to basic effective care for people with noncommunicable lung disease in Africa.

## Abstract (FR)

**CONTEXTE:**

Le poids mondial dû à l’asthme et aux bronchopneumopathies chroniques obstructives (BPCO) est substantiel et particulièrement important dans les pays à revenu faible et moyen, incluant de nombreux pays d’Afrique. Leur prise en charge est affectée par la disponibilité des tests de diagnostic et des mé dicaments essentiels. L’ étude a voulu explorer la disponibilité des services de spirométrie et de médicaments essentiels de l’asthme et des BPCO dans les pays d’Afrique.

**MÉTHODE:**

Des questionnaires ont é té distribués aux travailleurs de santé dans le cadre de la réunion annuelle de la *Pan African Thoracic Society Methods in Epidemiology and Clinical Research* (PATS MECOR) et de l’*International Multidisciplinary Programme to Address Lung Health and TB in Africa* (IMPALA). Les données ont été analysées par de simples statistiques descriptives.

**RÉSULATS:**

Ont été renvoyés 37 questionnaires représentant 13 pays d’Afrique. La disponibilité de la spirométrie a été de 73,0%. Les raisons les plus fréquentes de non disponibilité ont été le manque de connaissances relatives à l’utilité du test. Dans cet échantillon, 33,3% étaient confrontés à une indisponibilité sporadique due à des problèmes de maintenance. La disponibilité des médicaments essentiels est allée de 37,8% pour les inhalateurs de corticostéroïdes et de bêta-2 agoniste d’action prolongée à 100% pour un comprimé de 5 mg de prednisolone, l’indisponibilité étant surtout due à des problèmes de chaîne logistique.

**CONCLUSION:**

La disponibilité de la spirométrie est variable ainsi que celle des médicaments essentiels de l’OMS pour les BPCO et l’asthme dans les pays d’Afrique. Des stratégies sont requises afin d’améliorer l’accès à des soins de bases efficaces pour les patients atteints de maladies pulmonaires non transmissibles en Afrique.

## Abstract (ES)

**MARCO DE REFERENCIA:**

La carga de morbilidad mundial por asma y enfermedad pulmonar obstructiva crónica (EPOC) es considerable, en especial en los países de ingresos bajos y medianos, incluidos muchos países de África. En su tratamiento influye la disponibilidad de medios diagnósticos y medicamentos esenciales. En el presente estudio se examinó la disponibilidad de servicios de espirometría y medicamentos esenciales destinados al asma y la EPOC en países africanos.

**MÉTODO:**

Se presentaron cuestionarios a los profesionales de salud que asistieron a la conferencia anual MECORIMPALA (*Epidemiology and Clinical Research* e *International Multidisciplinary Programme to Address Lung Health and TB in Africa*) de la Asociación Torácica Panafricana. Los datos se analizaron mediante índices estadísticos descriptivos sencillos.

**RESULTADOS:**

Se recibieron 37 repuestas a los cuestionarios de 13 países africanos. La disponibilidad de la espirometría fue 73,0%. La razón más frecuente para su ausencia fue el desconocimiento de la utilidad de la técnica. En esta muestra, el 33,3% afrontaba una disponibilidad esporádica debido a problemas de mantenimiento. La disponibilidad de medicamentos esenciales osciló entre 37,8% para los inhaladores de la combinación corticoesteroides y β_2_-agonistas de acción prolongada y 100% para los comprimidos de 5 mg de prednisolona, debido en primer lugar a problemas en la cadena de suministros.

**CONCLUSIÓN:**

La disponibilidad de la espirometría y los medicamentos esenciales de la OMS para la EPOC y el asma es variable en los países africanos. Se precisan estrategias que mejoren el acceso a una atención básica eficaz de las personas con patologías respiratorias no transmisibles en África.

NON-COMMUNICABLE RESPIRATORY diseases (NCRDs) are a worldwide public health burden. The prevalence of asthma and chronic obstructive pulmonary disease (COPD) is increasing in most developing countries.^1^ However, as resources are inadequate, these diseases are neglected in many African counties, and there is therefore an urgent need to increase access to appropriate diagnostic and treatment facilities.^2^,^3^ Morbidity and mortality rates due to NCRDs are therefore high, adding to the existing burden of infectious diseases.^4^ Spirometry can be used objectively to diagnose airway disease, and is therefore a valuable diagnostic aid. From the small amount of literature on the availability of spirometry and the challenges of its implementation in Africa, it appears that availability is low.^5^ Similarly, the literature reports low availability across African countries of the essential medicines included in the WHO’s Model Essential Medicines List (EML).^5^

Diagnostic tests and medicines are needed for optimal management of patients with NCRDs and the reduction of morbidity and mortality in Africa. This study investigated the availability of spirometry services and the WHO-recommended essential medicines for asthma and COPD in African countries with the aim of defining the current situation and describing the challenges for implementing services, which could guide the development of future strategies to mobilise funding and improve availability.

## METHOD

### Design

This was a cross-sectional survey to explore the availability of spirometry and 15 different forms of medications from the 20th edition of the WHO EML that are essential for the management of chronic respiratory diseases.^6^

### Study population

Healthcare professionals who attended the PATS MECOR and IMPALA Conference in June 2019 were invited to complete the questionnaire during the conference. The inclusion criteria were all healthcare professionals who worked in an African healthcare facility and had knowledge about the availability of the diagnostic resources and medicines for asthma and COPD in their facility. Participants were physicians who worked in hospitals.

### Data collection

A structured questionnaire comprising closed and open-ended questions was developed. The questionnaire was piloted in the study. These were administered during the Conference and were self-completed by participants.

### Analysis

Results were analysed using simple descriptive statistics on Microsoft Excel (MicroSoft, Redmond, WA, USA). Responses from open-answer questions were grouped according to common responses.^7^ Some open-answer responses could not be grouped, and these were quoted directly.

### Approvals

Ethical approval was granted by the Liverpool School of Tropical Medicine (LSTM) Research Ethics Committee, Liverpool, UK. PATS gave permission for data collection at PATS MECOR. Participation was voluntary and non-incentivised.

## RESULTS

Thirty-seven questionnaires were completed by physicians representing 13 African countries. Nigerian participants represented 30% of the total surveyed (Figure).

**Figure:**
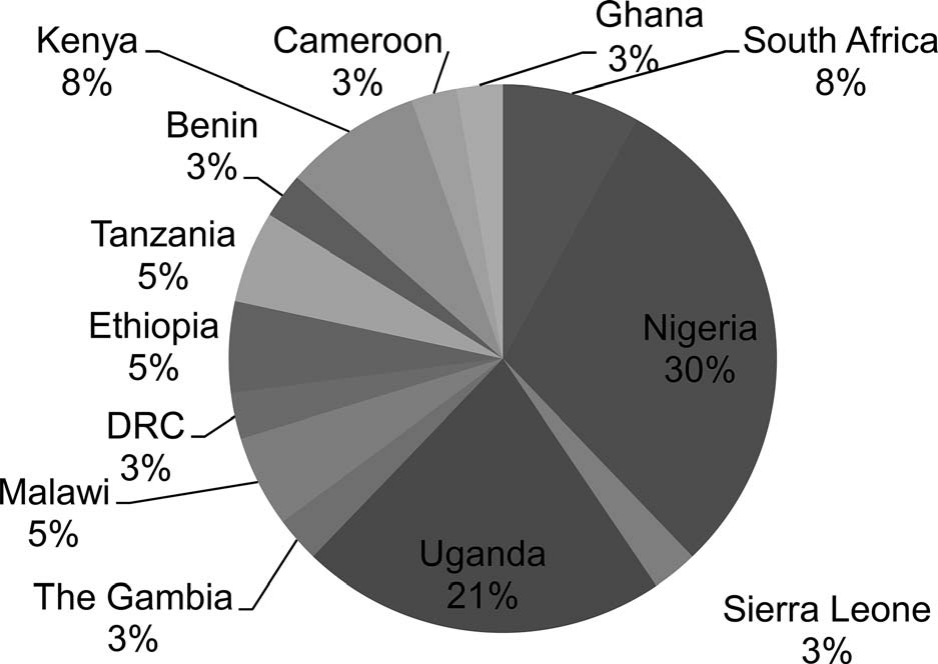
Pie chart showing countries represented by participants who completed the surveys. DRC = Democratic Republic of Congo.

### Availability of spirometry in African countries

A total of 37 (73%) participants reported having a spirometer at their facility, of whom 66.7% reported uninterrupted availability. Among those facilities with a spirometer, 77.8% of staff had training on operating the spirometer and 70.4% on data interpretation (Table 1). The most common reasons for unavailability was the belief that spirometry was not necessary or lack of training on how to use the spirometer (Table 2).

**Table 1:** Availability of spirometry, care of equipment and operator training received

	Spirometer available (*n* = 37) (%)	Spirometer available at all times (*n* = 27) (%)	Available spirometers that are calibrated (*n* = 27) (%)	Staff received training on the use of the available spirometer (*n* = 27) (%)	Staff received training on spirometry data interpretation (*n* = 27) (%)
Yes	73.0	66.7	59.3	77.8	70.4
No	27.0	33.3	29.6	11.1	18.5
Don’t know	0	0	11.1	11.1	11.1

**Table 2:** Reasons for why the spirometer was unavailable at the healthcare facility (multiple responses possible)

Reason for unavailable spirometer (*n* = 10)	Facilities reporting this reason (%)
Cost	20.0
No capacity in the facility	30.0
Not considered	50.0
No training for staff on how to use the spirometer	50.0
No training for staff on how to interpret the findings	40.0
No availability of disposals	30.0
Other	40.0

### Availability of WHO essential medicines for asthma and COPD

The medicine with the greatest availability was prednisolone 5 mg (100%), followed by salbutamol 100 mcg inhaler (94.6%). The least available medicines were ipratropium bromide 20 mcg inhaler (43.2%) and both doses of combination inhalers (38.7%) (Table 3).

**Table 3:** Availability of WHO essential medicines for asthma and COPD

WHO essential medicine	Medicine is available all the time (*n* = 37) (%)	Medicine is unavailable (*n* = 37) (%)	Availability of the medicine is unknown (*n* = 37) (%)	Of the medicines available (column 2), those which had interrupted availability (%)
Beclometasone HFA inhaler 50 mcg	59.5	29.7	10.8	45.5
Beclometasone HFA inhaler 100 mcg	64.9	24.3	10.8	45.8
Budesonide inhaler 100 mcg	48.6	40.5	10.8	44.4
Budesonide inhaler 200 mcg	48.6	40.5	10.8	44.4
Combination inhaler budesonide + formoterol 100/6	37.8	56.8	5.4	21.4
Combination inhaler budesonide + formoterol 200/6	37.8	56.8	5.4	21.4
Adrenaline 1 mg in 1 ml ampoule	89.2	10.8	0	6.1
Hydrocortisone powder for injection 100 mg in vial	89.2	8.1	2.7	9.1
Prednisolone 5 mg	100.0	0	0	2.7
Prednisolone 25 mg	81.1	16.2	2.7	3.3
Salbutamol inhaler 100 mcg (dry powder)	94.6	5.4	0	22.9
Salbutamol injection 50 mcg/ml in 5 ml ampoule	48.6	43.2	8.1	22.2
Salbutamol metered-dose inhaler 100 mcg	83.8	10.8	5.4	19.4
Salbutamol respirator solution for nebulisers 5 mg/ml	78.4	18.9	2.7	34.5

COPD = chronic obstructive pulmonary disease; HFA = hydrofluoroalkane.

### Payment modality for asthma and COPD medicines

For all medicines, the most common method of payment was full out of pocket pay by patients. Combination inhalers and ipratropium bromide were reported to be the most likely to be paid out of pocket. Salbutamol injections and prednisolone 25 mg tablets were most likely to be offered free of charge to patients. Rates of subsidised and mixed method payments were variable (Table 4).

**Table 4:** Payment modalities for asthma and COPD medicines in African countries

How is each available medicine paid for by patients	Free (%)	Subsidised (%)	Full out-of-pocket pay (%)	Mixed payment method (%)	Unknown (%)
Beclometasone HFA inhaler 50 mcg	18.2	22.7	40.9	18.2	0
Beclometasone HFA inhaler 100 mcg	20.8	25.0	37.5	16.7	0
Budesonide inhaler 100 mcg	16.7	16.7	50.0	17.0	0
Budesonide inhaler 200 mcg	16.7	22.2	44.4	16.7	0
Combination inhaler budesonide + formoterol 100/6	7.1	14.4	64.3	14.3	0
Combination inhaler budesonide + formoterol 200/6	7.1	14.4	64.3	14.3	0
Adrenaline 1 mg in 1 ml ampoule	27.3	15.2	45.5	12.1	0
Hydrocortisone powder for injection 100 mg in vial	27.3	18.2	42.4	12.1	0
Prednisolone 5 mg tablet	24.3	13.5	45.9	16.2	0
Prednisolone 25 mg	30.0	13.3	40.0	16.7	0
Salbutamol inhaler 100 mcg	17.1	17.1	48.6	14.3	2.9
Salbutamol injection 50 mcg/ml in 5 ml ampoule	33.3	0.0	55.6	11.1	0
Salbutamol metered-dose inhaler 100 mcg	16.1	12.9	58.1	12.9	0
Salbutamol respirator solution for nebulisers 5 mg/ml	24.1	6.9	51.7	17.2	0
Ipratropium bromide inhaler 20 mcg	6.3	12.5	68.8	12.5	0

COPD = chronic obstructive pulmonary disease; HFA = hydrofluoroalkane.

### Reasons for the unavailability of WHO essential medicines in healthcare facilities

Reasons for the unavailability were explored if a medicine was sometimes unavailable. Common reasons across all medicines included ‘cost’, ‘not on national EML’, ‘lack of training’, ‘supply chain problems’, ‘procurement challenges’, ‘out of stock’ and ‘limited prescription’. The main reasons for complete unavailability of all medicines were cost, the belief that the drug in question was not required and not being on the national EML; however, most facilities provided alternative free-text explanations (Table 5).

**Table 5:** Table to show explanations of why each WHO essential medicine was unavailable

WHO essential medicine	Cost (%)	Nowhere to store according to manufacturer’s instructions (%)	Not considered (%)	Not required (%)	Not on the national EML (%)	Lack of knowledge of prescription (%)	Other (%)
Beclometasone HFA inhaler 50 mcg (*n* = 11)	18.2	0	45.5	0	18.2	9.1	54.5
Beclometasone HFA inhaler 100 mcg (*n* = 9)	22.2	0	55.6	0	22.2	11.1	55.6
Budesonide inhaler 100 mcg (*n* = 15)	26.7	0	33.3	33.3	33.3	20.0	33.3
Budesonide inhaler 200 mcg (*n* = 15)	26.7	0	33.3	6.7	33.3	13.3	40.0
Combination inhaler budesonide + formoterol 100/6 (*n* = 21)	38.1	0	28.6	0	28.6	19.0	38.0
Combination inhaler budesonide + formoterol 200/6 (*n* = 21)	38.1	0	28.6	0	28.6	19.0	38.0
Adrenaline 1 mg in 1 ml ampoule (*n* = 4)	0	0	25.0	100.0	0	0	0
Hydrocortisone powder for injection 100 mg in vial (*n* = 3)	25.0	0	0	50.0	25.0	0	0
Prednisolone 25 mg (*n* = 6)	0	0	0	0	0	0	33.3
Salbutamol inhaler 100 mcg (*n* = 2)	0	0	100.0	0	0	0	0
Salbutamol injection 50 mcg/ml in 5 ml ampoule (*n* = 16)	12.5	0	25.0	12.5	37.5	12.5	37.5
Salbutamol metered-dose inhaler 100 mcg (*n* = 4)	50.0	0	100.0	50.0	0	0	25
Salbutamol respirator solution for nebulisers 5 mg/ml (*n* = 7)	14.3	14.3	57.1	0	28.6	28.6	14.3
Ipratropium bromide 20 mcg (*n* = 18)	16.7	0	27.8	5.6	44.4	5.6	22.2

EML = Essential Medicines List; HFA = hydrofluoroalkane.

### Other responses to explain drug unavailability

The most common alternative explanation for both doses of beclometasone hydrofluoroalkane (HFA) inhaler and combination inhalers being unavailable were ‘supply problems’. Other reasons for budesonide 100 mcg and 200 mcg inhaler being unavailable included ‘not cost-effective for pharmacies to store as drugs expire’ and ‘can be purchased at pharmacy shops by patients’. Prednisolone 25 mg tablets were unavailable because it was ‘not on the market’. Other reasons for salbutamol injection unavailability were ‘inhaled form is preferred’ and ‘not guideline-recommended’. One other reason provided for the unavailability of the salbutamol respiratory solution was ‘no nebuliser equipment at the facility’. Reasons for ipratropium bromide 20 mcg unavailability included ‘not currently available on the market, only nebuliser forms available’ and ‘not covered by insurance’.

## DISCUSSION

This study set out to evaluate the reported availability of spirometry services and WHO essential medicines at healthcare facilities where African participants attending a conference work in the context of the Global Action Plan for the Prevention and Control of NCDs 2013–2020, which has a target of ‘80% availability of the affordable basic technologies and essential medicines, including generics, required to treat major non-communicable diseases in both public and private facilities’.^8^

The main finding from this study is that there is modest availability of spirometry at health facilities across Africa, with very limited availability of WHO essential medicines, particularly inhaled steroids for the management of asthma and COPD. Nearly three quarters of participants had a spirometer at their institution, while only four essential medicines (adrenaline, hydrocortisone, salbutamol inhalers and prednisolone) reached the WHO target for availability.

The results are comparable to a 2009 survey of PATS MECOR members that showed that 62% of healthcare professionals used spirometry to diagnose COPD.^9^ However, a systematic review found that fewer than 30% of Ugandan and Nigerian healthcare facilities had access to spirometry services.^5^ A survey in Tanzania reported that only 1.9% of facilities in the study had the appropriate equipment (including spirometry) to manage NCRDs.^10^ Facilities in this study were more likely to have training to perform rather than interpret spirometry which risks misdiagnosis. A case study from Nigeria found that only 34% of respondents could perform full spirometry.^11^ About a third of facilities did not routinely calibrate the spirometer, suggesting results may be poor quality and incorrect. A previous review on dealing with spirometry access in Africa identified low availability of calibration equipment and technical support in facilities as a challenge. Local equipment providers are only available in a few African countries.^2^ A third of the facilities with a spirometer had interrupted access, highlighting issues of poor technical support and capacity. Spirometry was never available in 27% of facilities; this was generally because spirometry was ‘not considered’ and ‘there was no training on how to use the spirometer’. A review on the challenges of accessing spirometry found that South Africa was the only country providing formal spirometry training. Inadequate training leads to a lack of human resources and inaccurate results.^2^

Salbutamol inhalers were widely available but steroid monotherapy inhalers and combination inhalers had low availability. These results are similar to a systematic review of studies from Uganda, Sudan, Eritrea and Nigeria, which reported that salbutamol inhalers were available in 70% of the facilities included. In this review, combination inhalers were only available in 45% of healthcare facilities in only Uganda and Nigeria.^5^ Trends in the availability of salbutamol, steroid and combination inhalers in this survey are comparable to a study on the availability of medicines for asthma in tertiary hospitals in Nigeria.^12^ Beclometasone and budesonide were mainly unavailable, despite being listed as essential for the basic management of patients with these diseases. Supply problems were reported and it is noteworthy that budesonide inhalers were often not on most national EMLs. The lack of availability of steroid-containing inhalers is particularly worrying given their central role in asthma management and the recent Global Initiative for Asthma recommendations for the use of inhaled corticosteroid/formoterol as needed in mild asthma.^13^

Cost was the main factor affecting availability of combination inhalers. In a Nigerian study, the unavailability of these medicines was also linked to unaffordability.^12^ Ipratropium bromide had very low availability. This is in line with reports from a systematic review of facilities in Eritrea, Nigeria, Ghana, Uganda and Sudan—the highest availability in healthcare facilities here was 14.3%.^5^ In addition to being a COPD reliever, ipratropium bromide is recommended for asthma management in case of life-threatening exacerbations.^14^ Prednisolone had high availability and is likely to be used widely due to low cost. It should be noted that long-acting beta-agonists as stand-alone inhalers are not on the WHO EML, despite their importance in the management of COPD.^15^ We suggest that this should be considered for inclusion in the next WHO EML update. A common reason for inconsistent medicines availability was supply chain and stock issues which could relate to problems with manufacturing, product registration and distribution, among others. Absence from the national EML appears to affect the ready availability of medicines. A Ugandan survey noted that aminophylline tablets, beclometasone inhalers and salbutamol inhalers were the only essential medicines for asthma and COPD on the Ugandan national EML, impacting availability of other medicines.^1^ Full out of pocket pay was the main payment modality for all medicines. This raises the issue of affordability in NCRDs. A consequence of this may be that pharmacies would stop stocking medicines because of low demand. The Global Asthma Network (GAN) reported that medications essential for asthma treatment, especially steroid inhalers were often inaccessible in resource-poor settings.^16^

### Limitations of the study

Sampling PATS MECOR and IMPALA attendees may have resulted in over-reporting of availability as most were from urban, tertiary and often academic hospitals. Over-reporting was noted by Kibridge et al.^1^ and Mehrota, Oluwole and Gordon^9^ as a potential problem with survey delivery in African countries. Participants were mainly from Nigeria and Uganda, whereas the other countries generally had only one representative. Given the diversity in health systems and economies, solutions based on results may not be generalisable. Administering the surveys during a conference increased the likelihood of recall bias.

The majority of the questions were closed, which limited the number of explanations that respondents could provide to qualify their answers. Pre-defined options may have led respondents towards particular interpretations. For confidentiality purposes, names of the healthcare facilities and positions of participants were not included, although these could have been useful in interpreting responses. It is possible that some results were duplicated because a small number of attendees from Uganda and Nigeria may have answered with regard to the same healthcare facility. This did not become obvious in analysis, emphasising the risk of recall bias. This work provides a foundation for a larger-scale survey to comprehensively evaluate the availability of the basic essential medications for chronic respiratory disease services, including healthcare staff training, access and availability of diagnostics, and both pharmacological and non-pharmacological interventions for diseases prevention and control.

### Implications of study findings and recommendations

This study investigated continuous availability as well as quality of spirometry, in contrast to the previous survey administered at PATS MECOR where the focus was on spirometry usage. Training programmes to perform spirometry and interpretation of findings to ensure accurate results should be made more available and accessible, with monitoring of performance. PATS and Spirometry Training Services Africa (STSA) are already running training courses in several African countries.^17^,^18^ With greater awareness, these courses can grow, and funding can be raised to provide or subsidise the equipment needed.

Across African countries, there is a strong need to improve the availability of controller medications to ensure optimal management and quality of life of asthma and COPD patients. Combination inhalers are costly and strategies are needed to improve affordability. An update of the national EML in most African countries is needed urgently to include the most important medicines for treatment of these diseases and possibly eliminate medications such as oral salbutamol, which have a poor risk/benefit ratio.

## CONCLUSION

The study found a variable, but generally, low availability (below WHO targets) of diagnostic spirometry and essential medicines for asthma and COPD in 13 African countries, which was influenced by several factors. We highlighted important gaps that could be addressed to improve availability, which countries will need to address individually. Further research is required to evaluate the availability and barriers to access to affordable quality assured essential medicines and spirometers for NCRDs and to path solutions to the barriers.
